# Enhancing the Oxygen Evolution Performance by Introducing NiO‐Supported Mesoporous Titanium Dioxide

**DOI:** 10.1002/open.202500377

**Published:** 2025-10-10

**Authors:** Abdulrahman Y. Alzahrani, Mohammed A. Bahattab, Mohammed Mushab, Ganesh Kumar Anbazhagan

**Affiliations:** ^1^ Refining and Petrochemical Technologies Institute (RPTI) King Abdulaziz City for Science and Technology (KACST) P.O Box 6086 Riyadh 11442 Saudi Arabia; ^2^ Chemistry Department College of Science King Saud University Riyadh 11451 Saudi Arabia; ^3^ Centre for Global Health Research Saveetha Medical College and Hospitals Saveetha Institute of Medical and Technical Sciences Saveetha University Chennai 602 105 Tamil Nadu India

**Keywords:** alkaline solution, mesoporous, nickel oxide, oxygen evolution, titanium dioxide

## Abstract

Water electrolysis for hydrogen and oxygen production has considerable potential for sustainable development as a clean energy source.Herein, a controllable and reliable method is demonstrated for the doping of mesoporous TiO_2_ with nickel oxide *x*‐NTO for an efficient oxygen evolution reaction. An acetic acid‐assisted soft‐template method using block copolymer polyvinylpyrrolidone as a structure‐directing agent successfully synthesizes *x*‐NTO with uniform large mesopores. Compared to the pure TiO_2_ mesoporous (0‐NTO), all the *x*‐NTO exhibit greatly enhanced oxygen evolution reaction (OER) activity. Furthermore, the percentage of nickel oxide greatly impacts the OER performance of *x*‐NTO catalysts, and the ≈3.0 wt% (3.0‐NTO) shows the greatest activity for OER, due to a 0.270 V decrease of the onset potential, while demonstrating an overpotential (*η*) of 340 mV at 10 mA cm^−2^ in 1 M KOH solution and higher mass activity (66.50 mA mg^−1^). Likewise, the 3.0‐NTO electrode is very durable and engaged during electrolysis, without any obvious current declines or potential wandering seen during 12.0 h of electrolysis, confirming the 3.0‐NTO's potential to serve as an electrocatalyst in energy conversion technologies.

## Graphical abstract

Herein, a schematic representation of the synthesis of NiO‐supported mesoporous TiO_2_ is reported. The process involves self‐assembly of polyvinylpyrrolidone (PVP), Ti(acac)_2_, nickel salt, and ethanol, followed by solvent evaporation, thermal polymerization, and stepwise calcination under N_2_ (350 °C) and air (450 °C) to remove PVP and carbon, yielding the final mesoporous catalyst.

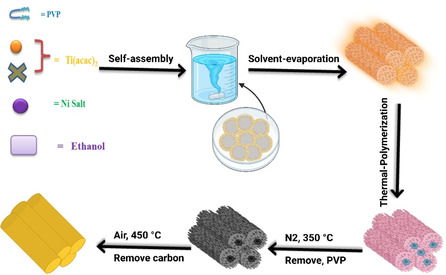

## 1 Introduction

Creating clean and sustainable energy supplies may be achieved by electrolysis or solar‐driven photoelectrochemical splitting of water to produce oxygen (O_2_) and hydrogen (H_2_) fuel, and this could prove to be a very attractive technology for overcoming our dependence on fossil fuels.^[^ ^1^ ^,^ ^2^ ^]^ A water electrolysis process in an alkaline media involves the oxygen evolution reaction (OER), which involves a thermodynamically uphill reaction requiring a high overpotential to occur.^[^ ^3^ ^,^ ^4^ ^]^ Nonetheless, the OER involves a slow reaction process and a relatively complex mechanism that limits its efficiency in water electrolysis.^[^ ^5^ ^,^ ^6^ ^]^ As a result, extensive studies and efforts have been devoted to developing efficient electrocatalysts for the promotion or acceleration of OER reactions. There is no electrode material that approaches the equilibrium potential of 1.23 V_RHE_,^[^ ^7^ ^,^ ^8^ ^]^ which represents the minimum energy required for the reaction to occur. Furthermore, noble metal oxides (e.g., RuO_2_ and IrO_2_) have been one of the most essential electrode materials because of their relatively high electrochemical properties in acidic environments.^[^ ^9^ ^]^ Therefore, electrocatalytic water splitting is not commercially feasible due to its high costs and scarcity. For this reason, it is pretty much mandatory for electrochemical OER to develop cost‐effective, stable, and reliable non‐noble metal electrocatalysts with efficient and durable performance. To avoid noble metals, transition metal oxides and hydroxides, such as nickel, cobalt, iron, molybdenum, titanium, and tungsten, have shown great potential as catalyst materials.^[^ ^10^, ^11^, ^12^, ^13^, ^14^ ^–^ ^15^ ^]^ However, it remains possible to improve the OER behavior of the transition metal oxides to make them more economically viable, stable, and suitable for commercial applications than noble metal catalysts. Among non‐noble metal OER catalysts, nickel oxide (NiO) is considered the best currently available non‐noble metal oxide catalyst, owing to its excellent catalytic ability and stability. However, NiO is constrained by its nature as a transition metal oxide, leading to poor conductivity and a limited number of active sites, which restricts its applicability in many areas.^[^ ^16^ ^]^ Given this, different NiO structures and characteristics should be studied to determine their effects on catalytic activity. For example, according to Babar et al.^[^ ^17^ ^]^ a porous NiO electrocatalyst with a nanowall structure was prepared using simple and cost‐effective thermal oxidation techniques on nickel foam substrates to improve OER efficiency. The NiO thin film had a low overpotential of 310 mV, resulting in a current density of 10 mA cm^−2^ and a lower Tafel slope of 54 mV dec^−1^ in a solution of alkaline electrolyte. Peitao Liu et al.^[^ ^18^ ^]^ presented a very efficient method for synthesizing a bifunctional electrocatalyst consisting of well‐defined porous Ni/NiO nanosheets. The Ni/NiO nanosheets electrocatalytic samples demonstrated remarkable electrocatalytic stability, exhibiting an extremely low overpotential of 1.49 V (10 mA cm^−2^) as well as a half‐wave potential of 0.76 V in OER and oxygen reduction reaction (ORR), correspondingly. Because of its porous nature, the material provides catalytically active sites as well as a favorable environment for both mass and charge transfer, leading to its outstanding electrocatalytic activity. Silva et al.^[^ ^19^ ^]^ used a green synthesis approach to fabricate NiO electrocatalyst with cubic nanostructure. The catalyst showed excellent electrochemical performance and stability when tested for OER in basic media. The NiO nanostructures were found to have a low overpotential of 307 mV to obtain *J* = 10 mA cm^−2^ as well as a small Tafel slope (60.35 mV dec^−1^). These studies have demonstrated that the pores in NiO (such as mesopores, nanosheets, hollow sphere structures, and so on) greatly increase the number of reactive sites. A common way of improving the conductivity of NiO is by using carbon materials to support it as a reaction catalyst.^[^ ^20^ ^]^ Carbon support, however, can be easily corroded by the electrolyte, resulting in a gradual degradation of its quality and a reduction of reaction stability. A recent study shows that TiO_2_ has excellent stability, environmental friendliness, and biocompatibility as catalyst support substrates.^[^ ^21^ ^,^ ^22^ ^]^ According to Chenhui Yang et al.^[^ ^22^ ^]^ they developed self‐supported Co@TiO_2_/Ti electrodes by anodic oxidation and hydrothermal reactions. As a substrate, TiO_2_ exhibits good corrosion resistance under OER conditions, and it provides a significant surface area for adding catalytically active Co catalysts. More recently, Xu et al.^[^ ^23^ ^]^ Synthesized Co_3_O_4_@TiO_2_ fibers using an electrospinning method and with a thermal annealing strategy. In their study, the researchers investigated how Co_3_O_4_ concentration affected the morphology of the TiO_2_ fiber surface, the crystal structure, the structure of the pores, the specific surface area, and the performance of the OER. The fibers with a Co/Ti molar ratio of 1:10 performed well both for OER and ORR reactions in alkaline solutions. Considering the local conductivity of the fiber, there was a small Tafel slope of 58 mV dec^−1^ as well as a low overpotential of 382 mV to provide a current density of 10 mA cm^−2^. Compared to carbon materials, transition metal oxides like TiO_2_ are more chemically stable and resist electrochemical corrosion, improving electrochemical catalysis. Additionally, mesoporous TiO_2_ provides more reactive surface sites, which, in turn, results in more mass transfer between reactants and products.^[^ ^24^ ^,^ ^25^ ^]^ Herein, we present a soft‐templating method using the evaporation induced self‐assemply method to prepare mesoporous nickel–titanium oxide (NiO/TiO_2_) with varying Ni weight percentages for the OER process. The 3.0‐NTO catalyst with 3 wt% exhibits the highest specific surface area of 247 m^2^ g^−1^ and an overpotential of 340 mV for OER (at 10 mA cm^−2^), comparable to the nickel‐based catalysts of previous studies.

## 2 Results and Discussion

### 2.1 Crystalline and Structural Properties of 0‐NTO and x‐NTO Samples

**Figure 1:**
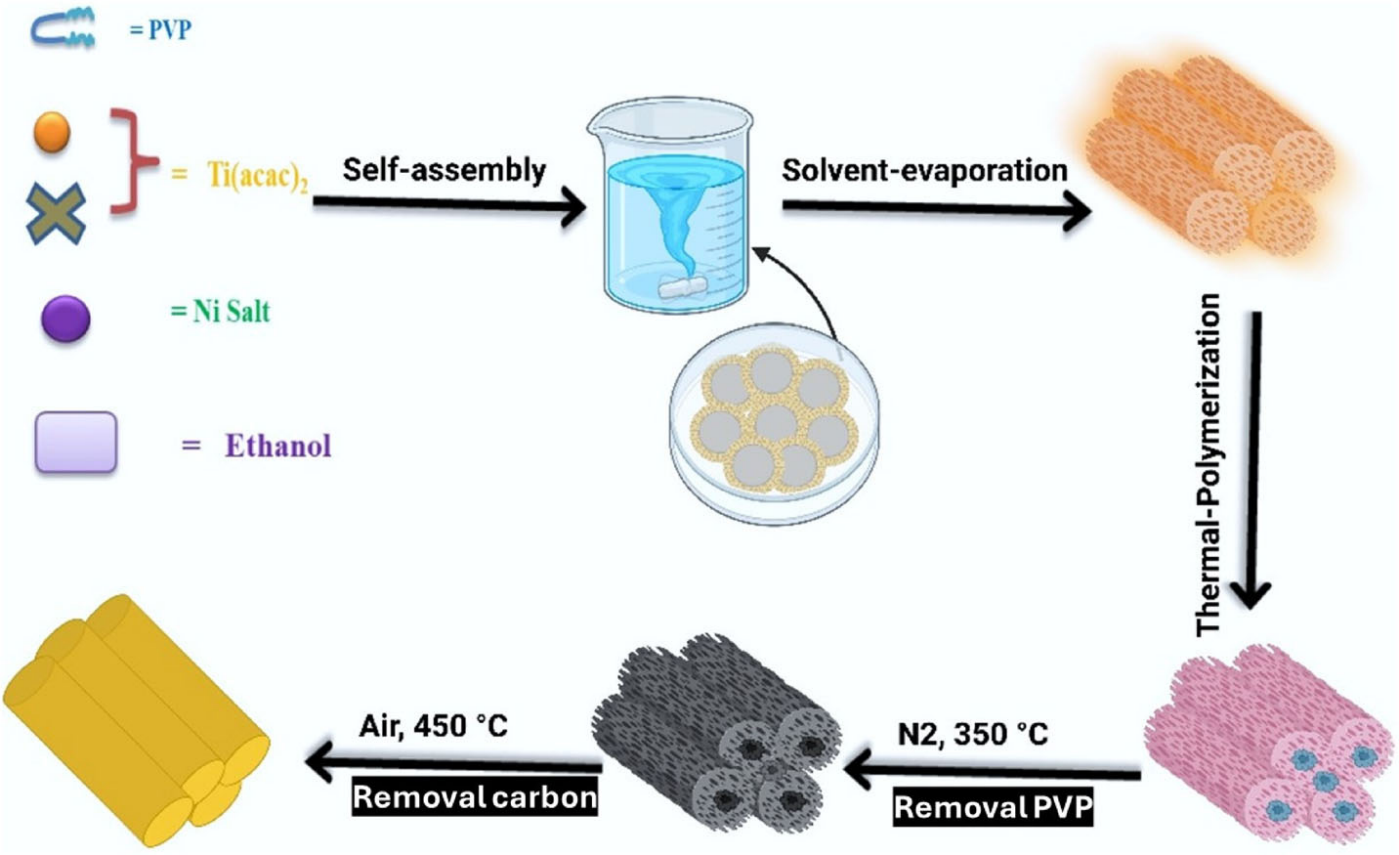
Schematic of the legand‐assisted soft‐templating synthesis of mesoporous Ni‐doped TiO_2_.

The schematic diagram in **Figure** **1** outlines the proposed pathway for the ligand‐assisted soft‐templating synthesis of Ni‐doped mesoporous TiO_2_. In the initial step, polyvinylpyrrolidone (PVP) acts as a structure‐directing agent, acetic acid functions as a chelating agent, and nickel nitrate and TOBT are utilized as the precursors for Ni and Ti, respectively. A composite micelle was formed when the acetic acid‐stabilized Ti precursor and the PVP block copolymers were evaporated from this solution. In an ethanolic solution, the hydrophilic segments of PVP were coassembled into rod‐like micelles along with Ni salt precursor. After solidifying at 90 °C for 24 h, the resulting composite membrane was scraped off and ground into a fine powder. This powder was then calcined at 350 °C for 3 h in N_2_ atmosphere and 450 °C in air for 3 h. Samples obtained were referred to as *x*‐NTO, with the *x* referring to the NiO weight ratio.

**Figure 2:**
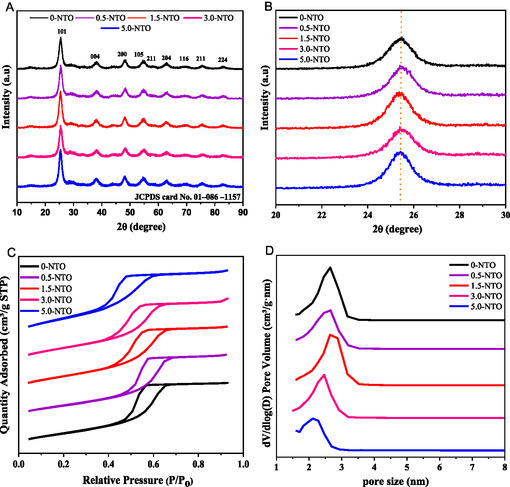
A,B) XRD patterns, C) N_2_ absorption–desorption isotherms, and D) pore size distributions of pure 0‐NTO and *x*‐NTO samples.

**Figure** **2** shows the effect of Ni amount on TiO_2_ crystal structure and N_2_ adsorption–desorption isotherms. A comparison of the X‐ray diffraction (XRD) spectra of *x*‐NTO samples with that of pure 0‐NTO mesoporous is shown in Figure 2A,B. As shown in Figure 2A, the XRD patterns reveal that the Ni‐doped TiO_2_ samples exhibit a highly crystalline anatase TiO_2_ phase (JCPDS card no. 01‐086‐1157), with peaks that closely match those of pure anatase TiO_2_. These findings indicate that the Ni dopant is uniformly distributed within the TiO_2_ matrix without altering the crystal structure of the TiO_2_ oxide. Moreover, the variation in Ni content of TiO_2_ mesoporous samples does not affect the crystallinity, and no NiO phase is detected on the surface of the mesoporous material. The (101) diffraction peak, magnified in pure 0‐NTO and *x*‐NTO mesoporous samples, showed no shift in 2*θ* values. However, the (101) diffraction peak remained the most intense in all samples, indicating that the primary crystal growth predominantly occurred along the same crystallographic plane of anatase TiO_2_,^[^ ^26^ ^]^ as shown in Figure 2B. To confirm the mesostructure of pure 0‐NTO and *x*‐NTO materials, the N_2_‐physisorption technique was employed (Figure 2C,D). As seen in all samples, the N_2_ adsorption–desorption isotherms have been shown to have type IV curves, accompanied by hysteresis loops and capillary condensation steps, which are all characteristic of mesoporous materials.^[^ ^27^ ^,^ ^28^ ^]^ A cylindrical mesoporous structure is visible in Figure 2C,D, which exhibits distinct capillary condensation within the relative pressure range (*P*/*P*_0_) of 0.4–0.8. Figure 2C,D illustrates the results of the Barrett–Joyner–Halenda (BJH) method in relation to the pore size distributions of TiO_2_ and Ni‐doped TiO_2_ samples. Additionally, **Table** **1** summarizes the textural parameters of TiO_2_ and Ni‐doped TiO_2_ materials derived from N_2_ adsorption–desorption isotherms and BJH analysis (Figure 2B,D). The results reveal that all samples possess excellent textural properties, including a high specific surface area (ranging from 215 to 247 m^2^ g^−1^), a large pore volume (between 0.2 and 0.35 cm^3^ g^−1^), and a uniform pore size distribution (2–2.7 nm). In addition to this, the incorporation of transition metals to the TiO_2_ mesoporous surface is known to decrease its surface area. Despite this, it has been found that the surface area of Ni‐doped TiO_2_ samples has been increased with the addition of transition metals, as compared with pure TiO_2_. This suggested that the introduction of Ni enhanced the porosity of the TiO_2_ mesoporous, resulting in an increased surface area and improved textural properties, which is beneficial for electrocatalytic processes, such as water oxidation.

**Table 1:** The BET surface area, pore volume, and average pore sizes of 0‐NTO and *x*‐NTO samples.

Samples	Molar ratio [Ni:Ti]	*S*_BET_ [m^2^ g^−1^]	Pore volume [cm^3^ g^−1^]	Pore size [nm]
0‐NTO	0	221	0.33	2.69
0.5‐NTO	0.0061	226	0.32	2.65
1.5‐NTO	0.018	233	0.35	2.33
3.0‐NTO	0.037	247	0.36	2.46
5.0‐NTO	0.061	215	0.32	2.12

Nevertheless, a modest decrease in Brunauer–Emmett–Teller (BET) surface area was observed for 5.0‐NTO compared to pure TiO_2_, which is attributed to the incipient aggregation and surface coverage of NiO nanoparticles that partially block access to smaller pores. Similar behavior has been reported in mesoporous TiO_2_ systems with increasing NiO loading, where excessive NiO leads to pore obstruction and reduced BET values.^[^ ^29^ ^,^ ^30^ ^]^

### 2.2 Surface Properties of 0‐NTO and x‐NTO Samples

**Figure 3:**
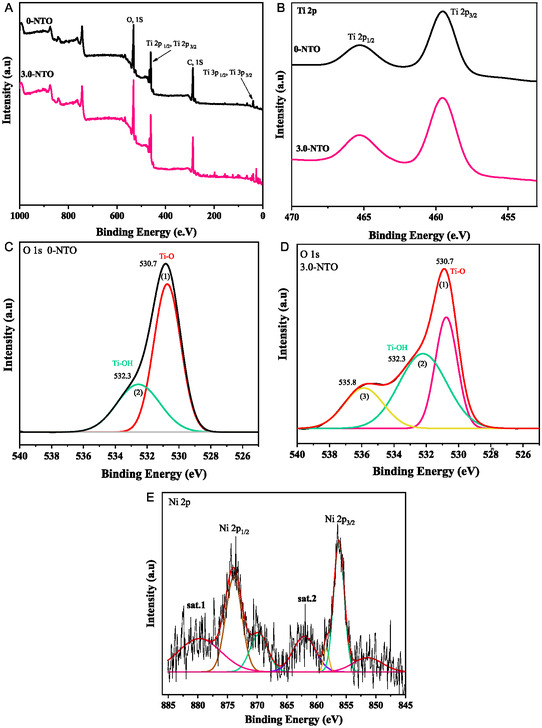
A) Wide spectra, B) Ti 2*p* XPS region spectra, C) O 1*s* region spectra of pure 0‐NTO, D) O 1*s* region spectra of pure 0‐NTO, and E) Ni 2*p* region spectra of 3.0‐NTO.

The comparison of X‐ray photoelectron spectroscopy (XPS) spectra of 3.0‐NTO composite materials and 0‐NTO is shown in **Figure** **3**. A comparative analysis of broad spectral data obtained from the XPS of 0‐NTO and 3.0‐NTO catalysts is presented in Figure 3A Some peaks agree with Ti 2*p* and O 1*s* in the XPS spectrum of 0‐NTO and 3.0‐NTO catalysts, but peaks that agree with Ti, O 1*s*, and Ni 2*p* are also demonstrated to exist in the 3.0‐NTO composites. In addition, the content of Ti, O, and Ni in composite materials was assessed to be ≈27.75, 71.44 and 0.81 at%, respectively (Table S1, Supporting Information). Pure TiO_2_ exhibits symmetric 2*p*_3/2_ and 2*p*_1/2_ photoelectron peaks.

These binding energies (BE) suggest the Ti^4+^ state at 459.55 and 465.29 eV, respectively.^[^ ^31^ ^,^ ^32^ ^]^ Ti^3+^ was also detected at 457.3 and 463 eV, consistent with Ti 2*p*_3/2_ and Ti 2*p*_1/2_, respectively.^[^ ^33^ ^]^ These two peaks have a spin–orbit splitting energy of 5.74 eV, which agrees with the values reported in the literature.^[^ ^34^ ^]^Upon doping Ni into TiO_2_, the 2*p*_3/2_ peaks of Ti in 1.5‐NTO samples are also comparable with those in pure TiO_2_, with a small redshift in BE of 0.15 eV, suggesting the displacement of Ti (IV) by Ni dopants.^[^ ^35^ ^,^ ^36^ ^]^ As with Ti^4+^, Ni^2+^ has a near‐identical ionic radius and could form the same octahedron coordination. There is, however, the possibility of replacing the Ti^4+^ ions with Ni^2+^ ions in the TiO_2_ lattice. Figure 3C,D shows XPS bands of the O 1*s* region of pure 0‐NTO and 3.0‐NTO samples. As revealed in Figure 3C, the O 1*s* spectrum of pure 0‐NTO reveals the presence of two distinct oxygen species. The experimental data were fitted using two Gaussian peaks, labeled as (1) and (2). Lattice oxygen from TiO_2_ and NiO oxides is attributed to the first peak (1) in the spectrum with a lower binding energy of 530.7 eV.^[^ ^37^ ^,^ ^38^ ^]^ There is a second peak, located at 532.3 eV, which corresponds to the OH groups adsorbing on the surface of mesoporous TiO_2_. In contrast, the O 1*s* region of the 3.0‐NTO sample revealed three Gaussian peaks, namely, (1), (2), and (3), which can be deconvoluted into three Gaussian peaks (Figure 3D). A peak energy of 530.7 eV is associated with the Ti—O bonds in TiO_2_ and NiO. While the other two peaks appearing at 532.3 and 535.8 eV are attributed to nonlattice or surface species (e.g., –OH and/or adsorbed H_2_O/CO_*x*_) groups on the sample surface.^[^ ^33^ ^,^ ^39^ ^]^ A high‐resolution scan of the Ni 2*p* spectrum is shown in Figure 3E. This figure shows peaks at 855.2 and 861.3 eV (satellite) relating to Ni 2*p*_3/2_, as well as a peak at 873.8 eV associated with Ni 2*p*_1/2_, indicating the presence of Ni^2+^ in the form of NiO.^[^ ^32^ ^,^ ^40^ ^,^ ^41^ ^]^ It is evident from the above discussion that the 1.5‐NTO catalyst consists of Ti^4+^, Ni^2+^, and O.

### 2.3 Morphological Features of 0‐NTO and 3.0‐NTO

**Figure 4:**
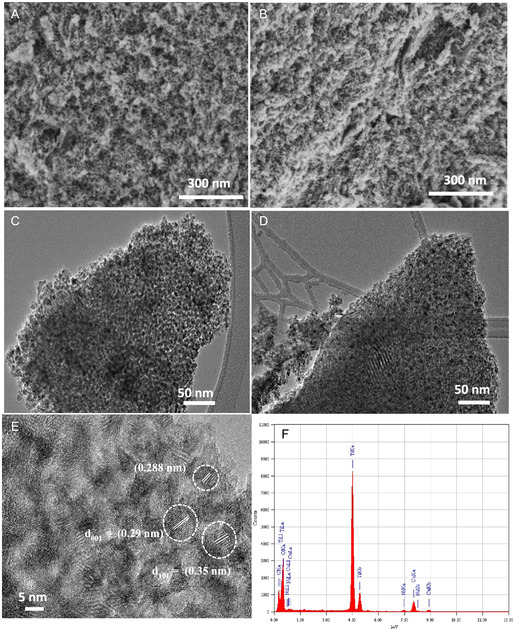
SEM images of pure A) 0‐NTO and B) 3.0‐NTO. TEM analysis of C) 0‐NTO and D) 3.0‐NTO sample. HR‐TEM analysis of E) 3.0‐NTO. EDX analysis of F) 3.0‐NTO.

**Figure 5:**
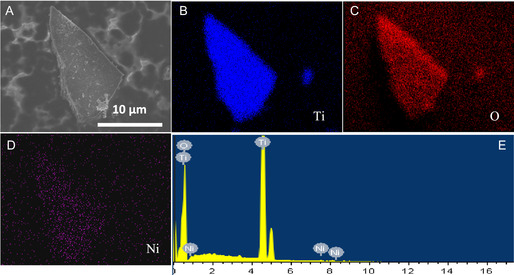
A–E) EDX elemental maps of Ti, Ni, and O. SEM and the EDX profile of Ti, Ni, and O elements of 3.0‐NTO.

Field emmision scanning electron microscope (FE‐SEM) and high resultion transmission electron microscope (HR‐TEM) analyses were conducted to inspect the morphology and mesostructural details of 0‐NTO and 3.0‐NTO. **Figure** **4**A,B shows the mesoporous structure of pure 0‐NTO and 3.0‐NTO catalysts, with well‐packed pores at short ranges and channels such as worms extending throughout. Figure 4C,D shows representative TEM images of pure 0‐NTO and 3.0‐NTO catalysts. A TEM image in Figure 4D clearly shows the hierarchically porous structure of 3.0‐NTO as the worm‐like structure containing a large connected smaller pore. According to Figure 4D, nickel oxide nanoparticles are represented by dark spots evenly distributed throughout the mesoporous TiO_2_ substrate. A high‐resolution TEM image of the 3.0‐NTO catalyst in Figure 4F shows crystalline structures with fringe spacing around 0.340 nm for TiO_2_ (101) plane diffraction. 3.0‐NTO is shown to be crystalline with fringe spacing around 0.340 nm for TiO_2_ (101).^[^ ^42^ ^,^ ^43^ ^]^ Nickel oxide nanoparticle's crystal lattice spacing is estimated at 0.243 and 0.288 nm, respectively, representing NiO diffraction planes (311) and (220).^[^ ^44^ ^]^ Upon incorporating 3.0 weight percent nickel, the nanoparticles aggregated into larger clusters, resulting in a disordered TiO_2_ substrate. This structural change is evident in the TEM images of the 3.0‐NTO catalyst, as shown in Figure S1, Supporting Information. The Ni‐doping concentration in the catalysts was quantified using energy‐dispersive X‐ray spectroscopy (EDX). The EDX‐mapping examination discloses the presence of the Ti, O, and Ni components through the whole structure of 3.0‐NTO (**Figure** 4F and **5**A–E). Interestingly, the EDX mapping reveals a uniform distribution of Ti, O, and Ni elements throughout the mesoporous framework. According to EDX analysis, the Ti, O, and Ni elements, individually, in the 3.0‐NTO catalyst have elemental compositions of 27.49, 67.32, and 3.05 wt%. It is important to note that EDX and XPS offer complementary insights into the elemental composition due to their different sampling depths: EDX reflects the overall (bulk) composition, whereas XPS probes only the top few nanometers of the surface. Accordingly, the comparatively lower Ni atomic percentage detected by XPS aligns with a relative depletion of Ni at the surface compared to the bulk.

### 2.4 Electrochemical Performance for OER

**Figure 6:**
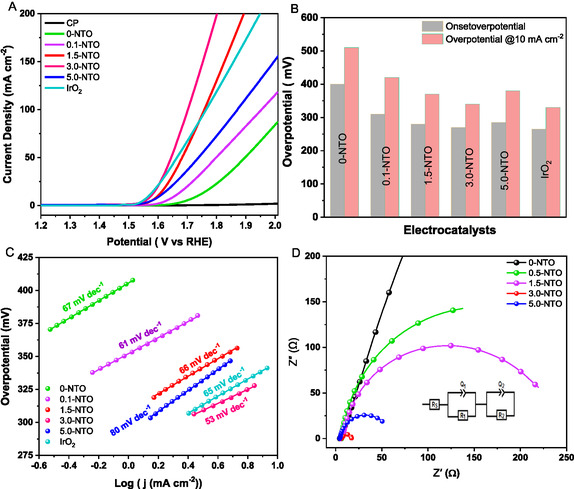
OER electrochemical tests. A) LSV profiles of *x*‐NTO and pure 0‐NTO catalysts in 1 M KOH at a scan rate of 10 mV s^−1^. B) Evaluation of the OER activity of the synthesized materials, including the OER onset potential and overpotential required to reach a current density of 10 mA cm^−2^. C) Tafel plots were obtained after analyzing the polarization diagram in (A). D) Nyquist plots of 0‐NTO and *x*‐NTO electrodes obtained at 1.55 V versus RHE in 1 M KOH solution.

A study of the electrocatalytic activity of the synthesized catalysts for the OER in alkaline media was evaluated by loading them onto carbon paper electrodes (1 cm^2^ diameter) and conducting linear sweep voltammetry (LSV), as illustrated in **Figure** **6**. The LSV curves of pure 0‐NTO, 3.0‐NTO, and commercial IrO_2_ catalysts are compared under identical testing conditions to evaluate their electrocatalytic performance. The LSV results reveal that the TiO_2_‐based catalyst demonstrates notable electrochemical activity in alkaline solutions. Compared to bare carbon paper (CP) electrodes, which do not display any catalytic activity for OER, the pure 0‐NTO catalyst, on the other hand, exhibits an OER overpotential of 505 mV at a current density of 10 mA cm^−2^. A significant increase in OER activity can be achieved by adding nickel oxide nanoparticles. The 3.0‐NTO catalyst demonstrates an onset voltage of 0.290 V versus referance hydrogen electrode (RHE) and a reduced overpotential (*η*) of 345 mV at 10 mA cm^−2^. This overpotential is 160 mV, far below that of pure 0‐NTO. The 3.0‐NTO mesoporous catalyst exhibits superior performance compared to 0.5‐NTO (428 mV), 1.5‐NTO (367 mV), and 5.0‐NTO (375 mV), all synthesized using the same method.

Moreover, its performance is comparable to that of commercial IrO_2_ (338 mV) and state‐of‐the‐art nickel‐based catalysts at the same current density, as shown in **Table** **2**.^[^ ^23^ ^,^ ^45^ ^–^ ^49^ ^]^ It has been observed that the electrocatalytic activity of *x*‐NTO is mostly affected by the doping concentration of Ni atoms within the TiO_2_ support. It is worth noting that the 3.0‐NTO‐loaded electrode was found to be the most effective electrode material for OER. A higher current density was observed when potential ranged from 1.52 to 1.65 V versus RHE. To further validate the electrocatalytic performance of *x*‐NTO electrode hybrids for the OER, key parameters such as mass activity and Tafel slope were evaluated, as summarized in Table 2. Electrocatalyst mass activity was determined according to the method described by Gao et al.^[^ ^50^ ^]^ At a potential of 1.6 V versus RHE, the mass activity of 3.0‐NTO reaches 66.50 mA mg^−1^. As evident from Table 2, the mass activity values of 3.0‐NTO significantly surpass those of the other investigated electrocatalysts, as well as previously reported values for state‐of‐the‐art catalysts.^[^ ^46^, ^47^ ^–^ ^48^ ^,^ ^51^ ^]^ As shown in Table 2, 3.0‐NTO catalysts have mass activities that are ≈8.98, 1.94, 2.46, and 1.09 orders of magnitude higher than those of 0.5‐NTO (7.40 mA mg^−1^), 1.5‐NTO (34.22 mA mg^−1^), 5.0‐NTO (27.04 mA mg^−1^), and IrO_2_ (61.10 mA mg^−1^), respectively.

**Table 2:** The OER activities of pure TiO_2_ and ni/meso‐TiO_2_ catalysts.

Catalyst	Onset *η* [V]	*η* [V] at 10mA cm^−2^	Mass activity at *η* = 0.35 V [mA mg^−1^]	Tafel slope [mV dec^−1^]	*R*_ct1_ [Ω]
*x*‐NTO	0.425	0.505	0.21	67	315.2
0.5‐NTO	0.359	0.428	7.40	61	31.20
1.5‐NTO	0.314	0.367	34.22	66	22.63
3.0‐NTO	0.290	0.345	66.50	53	9.37
5.0‐NTO	0.310	0.375	27.04	80	13.31
IrO_2_	0.284	0.338	61.10	65	9. 82

With increasing Ni content to 5.0 wt% in the 5.0‐NTO sample, the electrochemical performance slightly declines. This decline is attributed to NiO nanoparticle aggregation, as evidenced by TEM and HR‐TEM images showing particle clustering on TiO_2_ surfaces (Figure S1, Supporting Information). Minor NiO nanoparticles (highlighted with red circles) partially block active sites, reduce the effective surface area, and adversely affect the electrochemical activity. The superior OER activity of 3.0‐NTO is likely due to a combination of its unique structural properties (wormlike TiO_2_ substrate), the synergistic interaction between Ni and TiO_2_, and enhanced charge and mass transport capabilities. These factors collectively contribute to their high efficiency and stability as an OER catalyst. To further explore OER kinetics, a Tafel plot was constructed using the following equation: *η* = *b* log *j* + *a*, where *j* represents the current density and *b* is the Tafel slope.^[^ ^49^ ^,^ ^52^ ^]^ This can be seen in Figure 6C and Table 2, where the 3.0‐NTO catalyst exhibits the lowest Tafel slope (53 mV dec^−1^), thus making it the most productive of the studied and reference materials. To further quantify these observations, electrochemical impedance spectroscopy (EIS) was utilized to analyze the OER kinetics and charge transfer processes across all electrodes. Figure 6D shows distinct Nyquist plots of pure 0‐NTO electrode and *x*‐NTO electrodes combinations, measured at 1.55 V (vs RHE) with the impedance parameters shown in Table 2. The electrochemical impedance spectra were fitted using an equivalent circuit model comprising *R*_s_ (solution resistance), *R*_ct1_ and *R*_ct2_ (charge–transfer resistances), and a CPE (constant phase elements Q_1_ and Q_2_) representing the double‐layer capacitance), as illustrated in the inset of Figure 6D. As predicted, the lowest frequency zone and its analogous circuit correlate to the catalysts’ charge transfer resistance (*R*_2_). It was found that the values of charge‐transfer resistance (*R*_2_) decreased as NiO doped into the TiO_2_ lattices. In Table 2, the *R*_2_ values for 3.0‐NTO, 0.5‐NTO, 1.5‐NTO, 5.0‐NTO, and bare 0‐NTO are 9.37, 31.20, 22.63, 13.31, and 315.2 Ω, respectively. As compared to pure TiO_2_ and other studded electrodes, the *x*‐NTO electrode exhibits a smaller radius of the Nyquist curve, signaling reduced electron and charge transfer resistance, leading to faster electrode kinetics, conforming with its greatest natural activity regarding OER.

**Figure 7:**
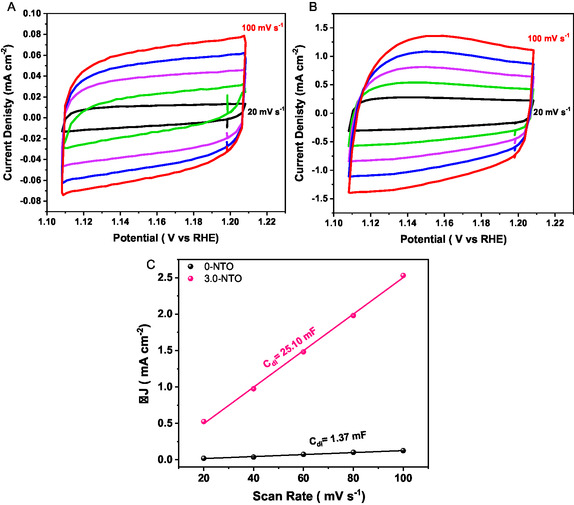
Electrochemical surface area measurements. Cyclic voltammograms of A) 0‐NTO and B) 3.0‐NTO at various scan rates. C) Linear plots of capacitive currents of the catalysts against scan rate.

**Figure 8:**
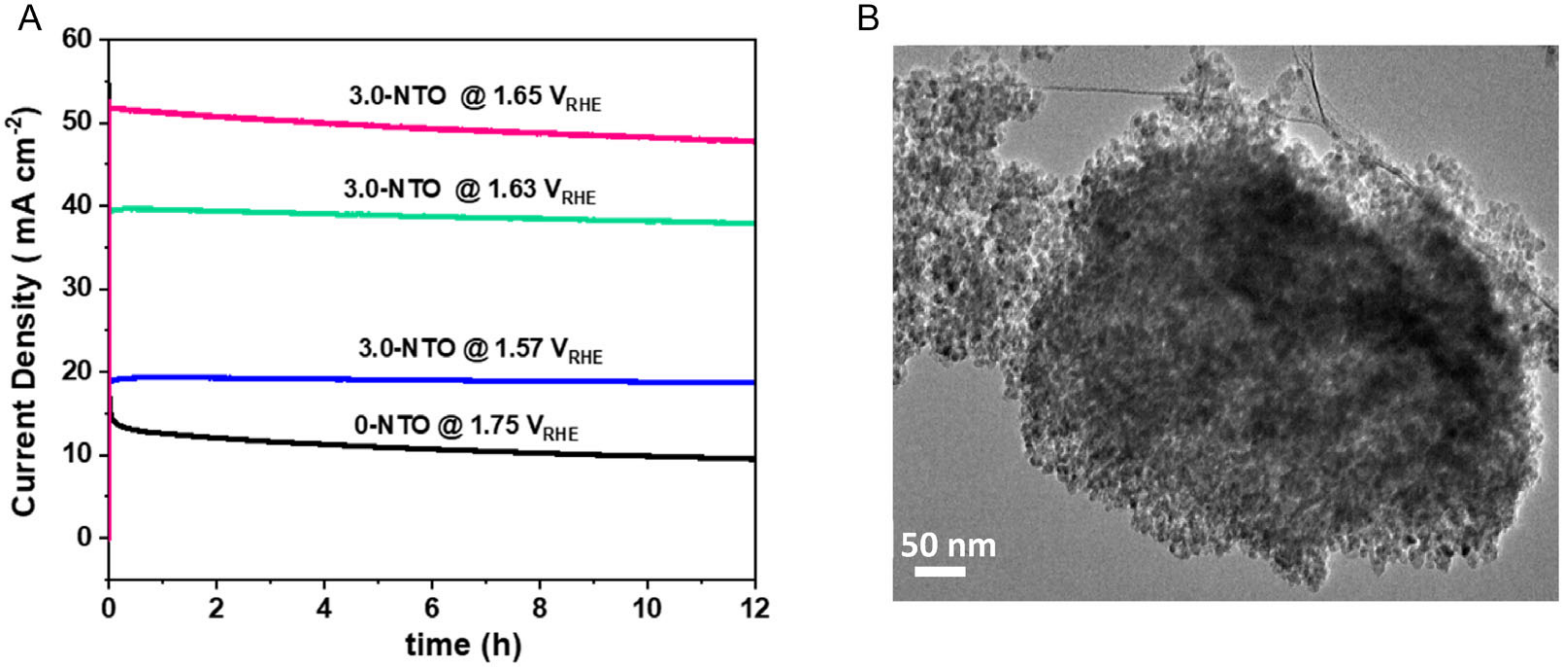
Long‐term operation tests. A) Chronoamperometry responses 0‐NTO and 3.0‐NTO catalysts taken in 1.0 M KOH under different potentials. B) TEM image of 3.0‐NTO catalyst after the chronoamperometry test.

The electrocatalytic performance of the prepared catalysts is closely linked to their electrochemical surface area (ECSA). The double‐layer capacitance (*C*_dl_), which is closely linked to the ECSA, was calculated utilizing the CV technique. **Figure** **7** shows *C*_dl_ evaluations in the non‐Faradaic area at different scan rates (20–100 mV vs RHE) for TiO_2_ (Figure 7A) and 3.0‐NTO (Figure 7B). Figure 7C plots the variation between positive and negative current densities (Δ*j*/2) versus scan rates, with the slope representing the *C*_dl_ value. A *C*_dl_ of 25.10 mF was achieved with 3.0‐NTO electrodes; however, the TiO_2_ catalyst exhibited a lower *C*_dl_ of 1.37 mF. Based on these findings, the 3.0‐NTO electrodes demonstrate improved electrocatalytic properties due to a greater number of exposed active sites. The long‐term durability of the catalyst is also crucial in determining its properties. To examine the catalyst durability for OER, the chronoamperometry measurement (*j*−*t*) for 0.NTO and 3.0‐NTO was carried out at different potentials in 1 M KOH (**Figure** **8**A). As demonstrated in Figure 8A, 0‐NTO needed 1.75 V versus RHE to achieve a current density of 15 mA cm^−2^ and saw a 64% decline in current density after 12 h of ongoing electrocatalysis in 1 M KOH solutions. On the other hand, for a 3.0‐NTO catalyst and at 1.57 and 1.63 V versus RHE, the 3.0‐NTO catalyst retains ≈90% of its initial activity after 12 h of continuous operation, indicating moderate stability with an ≈10% performance loss during the durability test. Additionally, the 3.0‐NTO catalyst undergoes no corrosion, surface passivation, or degradation when electrolyzed in alkaline solutions at a higher oxidation potential (1.65 V vs RHE), unlike some transition metal oxide catalysts.^[^ ^53^ ^,^ ^54^ ^]^

Following chronoamperometry testing, the loaded materials were carefully eliminated from the CP electrodes and analyzed with HR‐TEM (Figure 8B). The samples’ mesoporous nature is unchanged, and lattice fringes corresponding to the TiO_2_ and NiO crystalline structure are visible, indicating that the fabricated materials are structurally and crystallographically stable. A possible explanation for the OER activity of catalysts with 3.0‐NTO composition might be due to the synergy between the NiO and TiO_2_ mesopores. Furthermore, the higher specific surface area of the 3.0‐NTO electrode and the presence of disordered mesopores in the electrodes contribute to a higher density of charge carriers, this improves the activity of the electrocatalysis process.

## 3 Conclusions

We effectively produced exceptionally OER‐active, affordable NiO‐doped TiO_2_ mesoporous catalysts utilizing an interfacial soft‐template approach using an acetic acid‐assisted surfactant self‐assembly method. The influence of Ni‐doping levels on the morphological structure and electrocatalytic performance of NiO/TiO_2_ materials was extensively studied. Among the catalysts studied, the 3.0‐NTO material (with 3.0 wt% Ni doping) proved to be a very effective electrocatalyst for OER in an alkaline environment. This is demonstrated by its negative shift in onset potential to 1.52 V versus RHE, a low Tafel slope of 53 mV dec^−1^, and a high mass activity of 66.50 mA mg^−1^ at an overpotential (*η*) of 0.37 V versus RHE. In contrast to pure TiO_2_ (0‐NTO) catalysts, the 3.0‐NTO electrodes exhibit exceptional oxygen evolution activity and remarkable long‐term stability, comparable to benchmark transition metal oxide electrodes. The improved performance is due to the synergistic interaction between NiO and TiO_2_ mesopores, as well as the distinct structure of disordered mesopores inside a semicrystalline TiO_2_ framework. This structure provides a large surface area, abundant active sites, and a substantial solid–electrolyte interface, which collectively promotes efficient electron transfer and creates short diffusion pathways for reactants and products.

## 4 Experimental Section

4.1

4.1.1

##### Materials

The PVP (*M*_w_ = 1300,000) and titanium tetra‐butoxide were purchased by Aldrich Corp. Anhydrous ethanol (EtOH, 99.7%), hydrochloric acid (HCl, 37%), acetic acid (CH_3_COOH, 99.5%), and nickel nitrates were purchased from Alfa Aesar. CP (Fuel Cell Earth) was utilized as working electrode substrates, which were sequentially activated by acid treatment and then rinsed several times with deionized water. The rest of the chemicals were used as received.

##### Synthesis of TiO_2_ and NiO‐Doped TiO_2_ Mesoporous

Ni‐doped TiO_2_ mesoporous catalysts were prepared via the facial sol–gel in an ethanolic mixture of PVP, HCl, acetic acid (HOAc), and titanium tetrabutoxide (TBOT). Typically, 2g of PVP is dissolved in 30 mL of anhydrous ethanol coupled with 2.2 g of concentrated hydrochloric acid (37%). Following sustained stirring for 30 min, a transparent solution was produced. After that, a particular concentration of nickel nitrates was poured into the mixed solution, stirring continuously until a homogeneous, clear solution was achieved. Following this, 2.4 g of TBOT and 2.2 g of HOAc were included sequentially and stirred vigorously for 2 h to produce a homogeneous green solution. The obtained homogeneous green solution was then transferred to Petri dishes, where it was evaporated at room temperature for 20 min to remove all the solvents and then solidified at 90 °C for 24 h to remove all the solvents, resulting in the formation of hybrid inorganic–polymer compounds. To obtain crystalline *x*‐NTO oxide powder, the film was calcined for 3 h at 450 °C in air. The final products are called *x*‐NTO, where *x* refers to the NiO weight ratio. When the addition of nickel nitrate is 0 g, the sample is named 0‐NTO.

##### Material Characterization

Bruker D4 irradiation X‐ray diffractometer (Germany) was used to record wide‐angle XRD patterns under Cu Kα radiation (40 kV, 15 mA). TEM and HR‐TEM were performed using a JEM‐2100F microscope (Japan) operating at 200 kV. The FE‐SEM images were collected with a Hitachi Model S‐4800 microscope. Nitrogen adsorption and desorption isotherms were measured using the Micromeritics Tristar 3020 analyzer (USA). All samples were degassed under a vacuum at 180 °C for at least 6 h before measurement. For the specific surface area of the samples, the BET method was used, while the BJH method was used for pore diameter and volume distributions.

##### Electrochemical Measurements

Electrochemical measurements were performed with a three‐electrode setup using a VMP3 instrument (manufactured in France) with *x*‐NTO catalysts deposited on CP substrates as the working electrode, carbon graphite as a counter electrode, and Ag/AgCl as a reference electrode. The electrolyte used was 1 M KOH (pH = 14.0). Electrophoretic coating is employed to prepare the working electrodes, which consist of *x*‐NTO catalysts coated on CP. A solution of 10 mg iodine, Alfa‐Aesar, and 10 mg mesoporous sample powder was prepared with 20 mL acetone and sonicated for 1 h with an ultrasonic probe. The CP substrate (1 ×1 cm) was immersed in the solution at 1 cm^2^, and bias was applied for 4 min. After being rinsed with deionized water, the electrodes were dried in air and then calcined for 30 min at 300 °C under a nitrogen atmosphere. The mean weight of all mesoporous samples coated with CP was 0.3 mg. The EIS was carried out in a 1 M KOH electrolyte with a frequency of 10 kHz and an amplitude of 10 mV at 0.5 V versus Ag/AgCl.

## Conflict of Interest

The authors declare no conflict of interest.

## Author Contributions

**Abdulrahman Y. Alzahrani**: conceptualization, data curation, formal analysis, writing—original draft, investigation. **Mohammed A. Bahattab**: conceptualization, writing—review and editing, data curation, supervision. **Mohammed Mushab**: data curation, formal analysis. **Ganesh Kumar Anbazhagan**: writing—review and editing, data curation.

## Supporting information

Supplementary Material

## Data Availability

The data that support the findings of this study are available from the corresponding author upon reasonable request.
