# Supplementary material for: Enhancing the Oxygen Evolution Performance by Introducing NiO‐Supported Mesoporous Titanium Dioxide
Source: ChemistryOpen. 2025 Oct 10;15(4):e202500377. doi: 10.1002/open.202500377 (PMC13052062; doi:10.1002/open.202500377)
Supplement: Supplementary file 1 — Supplementary Material [file OPEN-15-e202500377-s001.pdf]

**Enhancing the oxygen evolution performance by introducing NiO-supported mesoporous titanium dioxide**

**Abdulrahman Y. Alzahrani**[a*], **Mohammed A. Bahattab**[a], **Mohammed S. Mushab**[b], **Ganesh Kumar Anbazhagan**[c,*]

[a]Refining and Petrochemical Technologies Institute (RPTI), King Abdulaziz City for Science and Technology (KACST), P.O Box 6086, 11442 Riyadh, Saudi Arabia

[b]Chemistry Department, College of Science, King Saud University, Riyadh 11451, Saudi Arabia.

[c]Centre for Global Health Research, Saveetha Medical College and Hospitals, Saveetha Institute of Medical and Technical Sciences, Saveetha University, Chennai – 602 105, Tamil Nadu, India.

# Supporting Information

Table S1. The atomic contents of Co, Ti, O in 0-NT0 and 3.0-NTO according to the xps reports.

| Sample | O /atom % | Ti /atom % | Ni /atom % | Ni/(Ni + Ti) /% |
|---|---|---|---|---|
| 0-NTO | 63.72 | 36.28 | 0 | 0 |
| 3.0-NTO | 71.69 | 27.44 | 0.81 | 2.87 |

**Table S2.** The relative atomic ratios of various elements in pure 0-NTO and x-NTO measured by EDX.

| Sample | Ti (%) | O (%) | Ni (%) |
|---|---|---|---|
| 0-NTO | 55.11 | 44.89 | - |
| 0.5-NTO | 40.49 | 59.38 | 0.14 |
| 1.5-NTO | 39.31 | 60.33 | 0.36 |
| 3.0-NTO | 39.31 | 60.33 | 0.36 |
| 5.0-NTO | - | - | - |

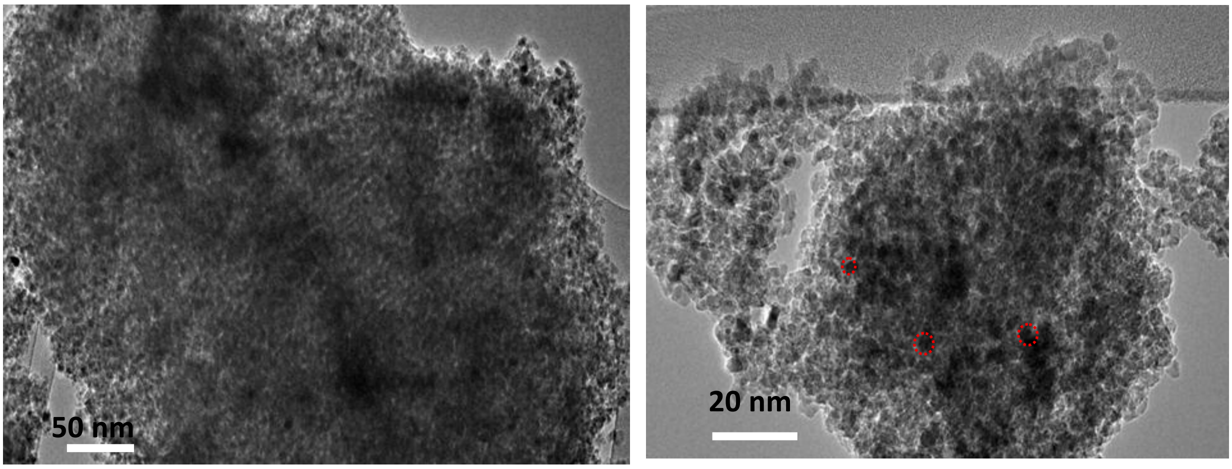


**Fig. S1.** TEM (a) and HRTEM (b) images of the 5.0-NTO sample, showing distortion and partial collapse of the mesoporous framework at high Ni loading. These structural changes are attributed to Ni aggregation and surface coverage, which reduce pore accessibility and adversely affect electrochemical performance.

**Table S3.** Nyquist plots derived from EIS measurements in 1.0 M $H_2SO_4$ at 1.55 V vs. RHE.

| Samples | $R_s$ (ohm) | $Q_2$ (F) | $Rct_1$ (ohm) | $Q_3$ (F) | $R_{ct2}$ (ohm) |
|---|---|---|---|---|---|
| x-NTO | 6.16 | 0.000604 | 315.2 | 0.000375 | 4.27 |
| 0.5-NTO | 6.03 | 0.00580 | 31.20 | 0.00160 | 0.335 |
| 1.5-NTO | 5.24 | 0.0062 | 22.63 | 0.000534 | 3.26 |
| 3.0-NTO | 5.26 | 0.00812 | 9.37 | 0.00673 | 0.84 |
| 5.0-NTO | 6.40 | 0.01166 | 13.31 | 0.00504 | 0.99 |

**Table S3.** Comparison of electrocatalytic activity and stability for different composite electrodes for OER.

| Catalyst | Substrate | Electrolyte | J(mA $cm^{-2}$) | η10 [mV] | Test condition | Stability [h] | mass activities (A $mg^{-1}$at η = 370 mV) | Reference |
|---|---|---|---|---|---|---|---|---|
| 3.0-NTO | CP | 1 M KOH | 10 | 345 | 1.570 | 12 | 66.50 | This study |
| Cobalt-Doped Black $TiO_2$ NAs | Black NTA | 1M KOH | 10 | 352 | 1.582 | 200 | - | 49 |
| NiFe/N-$TiO_2$ | N-$TiO_2$ | 1M KOH | 10 | 235 | 1.482 | 12 | - | 50 |
| Ni-doped $CoS_2$/NGA | GC (RDE) | 1M KOH | 10 | 330 | 1.60 V | 7 | - | 51 |
| $V_2C$-$TiO_2$ | GC | 1M KOH | 10 | 425 | 50 mA $cm^{-2}$ | 48 | - | 52 |
| CT-10-Ar | GC | 1M KOH | 10 | 480 | 10 mA $cm^{-2}$ | 16 | - | 53 |
| $Co_3O_4$@$TiO_2$ | GC | 1M KOH | 10 | 382 | - | - | - | 54 |
| | GC | 0.1M KOH | 10 | 308 | 1.53 V | > 2 | 54.9 @ 350 mV | |
